# Determining a sampling regime for PCR detection of respiratory tract viral infection at coronial post-mortem examinations

**DOI:** 10.1007/s12024-020-00273-w

**Published:** 2020-06-23

**Authors:** Caitlin Gilsenan-Reed, Geoff Higgins, Neil Langlois

**Affiliations:** 1 School of Medical and Health Sciences, University of Adelaide, Adelaide, South Australia Australia; 2 Microbiology and Infectious Diseases Directorate, SA Pathology, North Terrace, Adelaide, South Australia Australia; 3 Consultant Forensic Pathologist, Forensic Science SA, GPO Box 2790, Adelaide, South Australia 5001 Australia

**Keywords:** Post-mortem, Virology, Sampling, Nasopharyngeal, Tracheal, Lung, Respiratory tract

## Abstract

Death due to respiratory infection is commonly encountered at autopsy. With only one opportunity to obtain samples for identification of a causative agent, it is important to ensure that sampling regimes are optimized to provide the greatest detection, without the expense and redundancy that can arise from over-sampling. This study was performed retrospectively using data from Coronial autopsies over the period 2012–2019 from which swabs from the nasopharyngeal region, trachea and lung parenchyma, in addition to samples of lung tissue, had been submitted for multiplex PCR detection of respiratory pathogens. From 97 cases with all four samples, there were 24 with at least one positive result for viral infection. Some cases had multiple positive results and a total of 27 respiratory tract viruses were identified, of which rhinovirus, influenza A virus and respiratory syncytial virus were the most common. Seventeen of the 27 viral infections (63%) were identified in all four samples. However, in nearly all cases (96%) the nasopharyngeal swab detected the infective agent when the multiplex PCR panel had detected infection in any of the four sample types. A nasopharyngeal swab is considered to be an optimal sample for detection of respiratory tract viral infection. As the samples analyzed were acquired before the appearance of the COVID-19 virus, the applicability of this finding for COVID-19 screening is not established.

## Introduction

Lower respiratory tract infections are the third largest cause of death globally, so it is unsurprising that deaths following suspected respiratory infections are often referred to the Coroner [1]. In such cases, microbiological investigations can allow determination of the responsible organisms. Furthermore, in the setting of epidemics and pandemics, it is pivotal from a public health perspective to ensure that there is accurate detection of notifiable conditions to inform monitoring, as well as the continued observation for emerging conditions [2]. At autopsy, there is only one chance to obtain samples to identify infection. As there is a lack of standardization relating to sample collection post-mortem, there is variation pertaining to the number and nature of samples collected for microbiological investigation at autopsy [3]. Without guidelines to inform best practice for detection of respiratory tract infection, multiple swabs may be taken alongside lung tissue to minimize the risk of missing infection. This can equate to both a high cost for the investigating institution, and increased workload for pathology centers. There is potential to mitigate this issue by determining which sample yields the greatest number of positive results for respiratory viral infection. The notion of the “optimal sampling regime” for respiratory illnesses was explored by Moore and Jones in 2014, wherein they arrived at the conclusion that swabbing is more accurate than tissue sampling, as influenza and other respiratory viruses were more readily detected [4]. This study sought to explore the practice of sampling for detecting respiratory tract viral infection with the intention to find an optimal sample.

## Materials & methods

Cases were sourced from deceased who had undergone full internal post-mortem examination in South Australia at the direction of the Coroner from the period of June 2012 to June 2019 wherein respiratory samples were taken to assess for the presence of infective agents. Such cases were identified by searching Coronial autopsy reports held at Forensic Science SA for results of respiratory viral investigation. This yielded 304 cases; however, all four respiratory samples (nasopharyngeal swab, tracheal swab, lung swab, and lung tissue) were only taken at autopsy in 97 cases. Of these, 24 cases were reported positive for a respiratory virus although some detected more than one virus. Cases that detected non-viral pathogens were not included in this study. Ethics approval for the project was provided by the University of Adelaide HREC (ref H-2018-146).

Nasopharyngeal swabs were taken by inserting the swab into each nasal orifice directly posteriorly, parallel to the floor of the base of the skull, to the posterior wall of the nasopharynx in turn. Tracheal swabs were obtained by directly swabbing the trachea once it had been removed with the thoracic pluck. Lung samples were obtained after the pleural surface had been sterilized using alcohol swabs (to enable the samples to also be suitable for bacterial culture). The pleura was incised and a sample (around 0.2–0.5 × 0.5 × 0.5–1.0 cm) was taken from the underlying lung tissue from the lower lobe of each lung. A swab was inserted into the incised defect and pressed into the lung parenchyma to obtain the lung swab. All swabs (QSwab, Delta Laboratories, Australia) came with viral transport medium into which the swabs were immersed and sealed for transport. Lung samples were transported dry in a sealed sterile container.

All respiratory tract swabs were analyzed at SA Pathology using a laboratory developed respiratory viral and bacterial multiplex PCR panel. The panel was able to detect 9 viruses (Adenovirus, Human metapneumovirus, Influenza A and B, Parainfluenza 1, 2 & 3, Rhinovirus and Respiratory Syncytial Virus) and 2 bacteria (*Bordetella pertussis* and *Mycoplasma pneumoniae*). Total nucleic acid was extracted from 200 μl of the swab virus transport medium using the Roche MagNA Pure 96 DNA and the Viral NA Small Volume Kit on the Roche MagNA Pure platform and eluted into 100 μl. Real time PCR reactions were performed on the Roche LC480 thermocyclers in 12.5 μl reactions using the Invitrogen SuperScript III Platinum One-Step qRT-PCR Kit 2× mastermix (for all reactions) and 2.5 μl of extracted nucleic acid. Lung biopsies were first processed by beating with glass beads in 2 ml of Roche External Lysis buffer for 20 min and then 200 μl was used for nucleic acid extraction.

## Results

We found 94 cases that had been autopsied between June 2012 and June 2019 from which four respiratory samples (nasopharyngeal swab, tracheal swab, lung swab, and lung tissue) had been obtained for testing by multiplex PCR panel. From these, there were 24 cases that reported at least one positive virology result. Not included in this study were two cases in which Mycoplasma pneumoniae was reported in all four samples. Of the cases with viral infection, the mean age was 48.4 years (median 56 years; interquartile range 29–72.8 years). Just over half the cases were female (13/24; 54%) (Table 1).

**Table 1:** Table of sex, age, viruses identified and cause of death of 24 cases identified over the period 2012–2019 for which four samples (nasopharyngeal, tracheal and lung swabs as well as lung tissue) had been submitted for multiplex PCR for viral respiratory tract pathogens with at least one positive result. (RSV = Respiratory Syncytial Virus)

Case no.	Sex	Age	Virus	Cause of death
1	F	35	Influenza A	Influenza A infection
2	F	75	Rhinovirus	Pneumonia
3	M	43	Parainfluenza 3	Pneumonia with mixed drug toxicity
4	M	39	Respiratory Syncytial Virus	RSV infection
5	F	<1	Rhinovirus	Undetermined
6	F	82	Respiratory Syncytial Virus	Ischemic heart disease, pneumonia and renal failure
7	F	27	Influenza A, Parainfluenza 1	Sepsis, pneumonia, influenza A virus
8	F	36	Influenza A	Pneumonia with mixed drug toxicity
9	M	61	Human metapneumovirus	Respiratory tract infection
10	F	88	Rhinovirus	Pneumonia
11	M	73	Influenza B	Pneumonia (influenza B and *Streptococcus pneumoniae*)
12	M	72	Respiratory Syncytial Virus	Pneumonia and cardiomegaly
13	M	61	Respiratory Syncytial Virus	Pancreatitis and RSV infection in a man with cardiomegaly
14	M	51	Influenza A	Respiratory tract infection with pneumonia
15	F	<1	Rhinovirus	Unascertained
16	F	37	Rhinovirus	Unascertained
17	F	74	Rhinovirus	Respiratory tract infection complicating COPD
18	M	67	Respiratory Syncytial Virus	Infective exacerbation of COPD
19	M	74	Respiratory Syncytial Virus	Cardiomegaly with RSV infection complicating emphysema as a possible contributing factor
20	F	1	Adenovirus, Parainfluenza 3, Rhinovirus	*Pseudomonas aeruginosa* sepsis
21	F	72	Influenza A	Influenza A infection
22	M	26	Rhinovirus	Compression of the neck consistent with hanging
23	M	69	Rhinovirus	Pneumonia
24	F	<1	Rhinovirus	Unascertained

A total of 27 respiratory pathogens were detected from the 26 cases. After rhinovirus (*n* = 10, 37%), the next highest detection rates were for influenza A (*n* = 5, 19%) and respiratory syncytial virus (RSV) (*n* = 6, 22%). It was noted that RSV was more common in those over the age of 60, but this was not statistically significant (83% of cases; chi square *p* > 0.05). However, the reverse trend was observed for influenza A, where 80% of cases were detected in those under the age of 60 (not statistically significant: chi square p > 0.05). Parainfluenza 3 was reported twice (7.4%). Adenovirus, human metapneumovirus, influenza B and parainfluenza 1 were only observed on one occasion each (3.7%). Neither bordetella pertussis nor parainfluenza 2 were detected. Twenty-two of the 24 cases were positive for one virus only (92%); one (case 7, Table 1) was positive for two viruses and in one case (case 20, Table 1) three viruses were detected.

The presence of virus was reported in all four samples (nasopharyngeal swab, tracheal swab, lung swab and lung samples) in 17 of the 27 reported viruses (63%). Of the cases where the virus was not detected in all four samples, it appeared only in the swabs. There was never a case where a virus was detected in the lung tissue alone. A lung tissue result was only ever positive when all other samples taken were also positive (Tables 2 & 3).

**Table 2:**
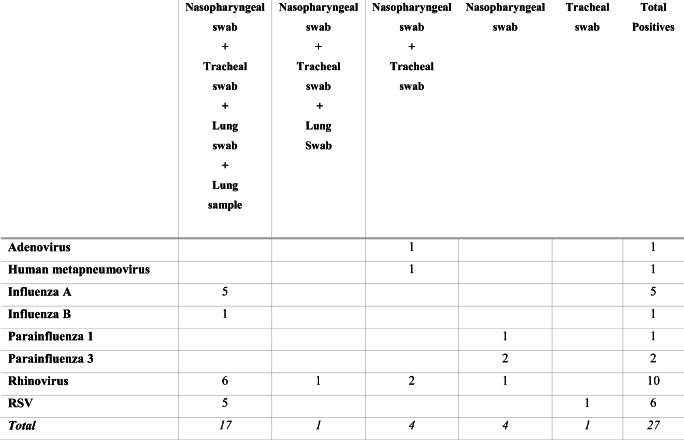
Detection rates for the combinations of samples in cases with four samples (nasopharyngeal, tracheal and lung swabs as well as lung tissue). Data from 24 cases with 27 reported viral respiratory pathogens from multiplex PCR panel. (Sample combinations which did not return results in a case, i.e. “lung tissue only” or “nasopharyngeal, tracheal and lung swabs” have been omitted.) (RSV = Respiratory Syncytial Virus)

**Table 3:** Frequency and proportion of viral detection per sample type. Data from 24 cases with four samples (nasopharyngeal, tracheal and lung swabs as well as lung tissue) submitted for multiplex PCR, which detected 27 respiratory tract viral infections

	Nasopharyngeal swab	Tracheal swab	Lung swab	Lung tissue
Adenovirus	1	1	0	0
Human metapneumovirus	1	1	0	0
Influenza A	5	5	5	5
Influenza B	1	1	1	1
Parainfluenza 1	1	0	0	0
Parainfluenza 3	2	0	0	0
Rhinovirus	10	9	7	6
RSV	5	6	5	5
Total Positive Samples	26/27 (96%)	23/27 (85%)	18/27 (67%)	17/27 (63%)

Most notably, the nasopharyngeal swab had the greatest respiratory infection pick-up with detection of 26 of the 27 viruses identified (96%). The nasopharyngeal swab did not detect one case of respiratory syncytial virus that was detected by a tracheal swab (which was considered a possible contributing factor to the death – see case no. 15, Table 1). Lung tissue had the poorest detection rate, only picking up 17 of 29 respiratory infections (63%) (Table 3).

The viral infection detected was listed in the cause of death in 7 of the 24 cases and there were a further 11 cases that denoted pneumonia or respiratory tract infection in the cause of death without naming the causative organism (Table 1).

## Discussion

As the respiratory tract is a common site of infection, it is an important component of the post-mortem examination to be able to accurately detect the causative microorganisms [1]. Key findings from the results of this study using retrospective data from Coronial post-mortem examinations are (1) of all the samples used, nasopharyngeal swabs yielded the greatest proportion of respiratory tract viral infection and (2) lung tissue alone was not an optimal specimen for detection of respiratory tract viral infection by multiplex PCR.

The prevalence of viruses detected was not unexpected. Rhinovirus, which is most commonly associated with the “common cold” was the most common. Whilst it is considered to cause a relatively benign illness, it is implicated in the exacerbation of respiratory diseases including chronic obstructive pulmonary disease, asthma and cystic fibrosis [5]. However, it is difficult to comment on the role of rhinovirus in this study, as in half of the cases when rhinovirus was detected, the cause of death was either unascertained, or considered to be due to a non-infective cause. Respiratory Syncytial Virus (RSV) and influenza were the next most common viruses that were identified [6]. It was noted that influenza A was more commonly seen in those under 60, and vice-versa for RSV, but this was not statistically significant. The observation may be attributed to the high influenza vaccination rates of the elderly in Australia and the fact that RSV increasingly affects the elderly, both in care facilities and the community [7].

For the optimal sampling regime at autopsy, it has been previously suggested that a minimum of four samples should be taken in cases of sudden-unexpected death with respiratory symptoms at any age, namely: lung, nasopharyngeal swab, bronchial swab, and at least one other swab from the affected tissue [2]. In addition, throat swabs are recommended when tonsillitis is apparent, and when empyema is suspected, it is recommended to sample pleural fluid [2]. Whilst this certainly offers the greatest opportunity for maximal detection of an infectious agent, it could be argued that there is a redundancy when it is possible to detect the overwhelming majority of respiratory tract viral infection using only a nasopharyngeal swab. Nasopharyngeal swabs have been shown to have a high yield of detecting viral respiratory tract infection [8]. Due to the mechanics of the mucocillary escalator system, the airways are more likely to contain downstream pathogens contributing to disease, hence making the nasopharyngeal area a prime location for collection [9]. The nasopharyngeal swab has previously been advocated in proposed guidelines relating to sampling in the setting of suspected flu or viral respiratory infection [3]. Certainly, the results of this study serve as confirmation of this strategy. This study was performed before the severe acute respiratory syndrome coronavirus 2 (SARS-CoV-2) pandemic due to corona virus (COVID-19) infection. Nonetheless, the findings support the current United States of America Centers for Disease Control and Prevention recommendation for post-mortem investigation, which is to submit a nasopharyngeal swab for virological testing with a lung swab if an autopsy is performed [10]. Nasopharyngeal swabs have been advocated for post-mortem sampling for COVID-19 [11] with positivity from nasopharyngeal swabs and failure to identify COVID-19 from lung swabs being reported [12]. Incorporating bronchial swabs has also been suggested [11, 13]. Others, including the Royal College of Pathologists [14] suggest using a strategy based on testing in living subjects, including use of sputum and bronchoalveolar lavage samples [15]. A further consideration could be use of rectal swabs [16]. It is not possible to comment on the potential utility of samples others than those used in this reported study.

It was considered that it might be possible to minimize the number of samples taken and consequently reduce costs, by obtaining a sample of lung tissue for the purpose of both multiplex PCR viral and bacteriological culture study. However, the results indicate this would not be ideal, as lung samples yielded the poorest results overall, only detecting 63% of viruses. Nonetheless, even with a lower detection rate, all respiratory tract viral infections regarded as notifiable by the State’s Communicable Disease branch [17] would have been identified by analysis of a sample of lung. One of the main issues with microbiological investigations on lung tissue is that in cases of focal infection, there is a possibility of generating false negative results if the sampling is undertaken outside of the affected region, which could explain the low viral yield [18]. It was also considered the higher yield from lung swabs might reflect that these were bathed in viral transport medium compared with the lung samples that were not. Further investigation would be required, but studies, including for a possible effect of viral transport medium, were outside the scope of this study.

In this study that retrospectively reviewed 94 cases, from which four respiratory tract samples (nasopharyngeal swab, tracheal swab, lung swab, and lung tissue) had been obtained for multiplex PCR viral detection, it was found that a multiple sample strategy detected only one case of viral infection that would not have been reported from a nasopharyngeal swab alone. Although this was a small, retrospective study with only 27 viruses identified from 24 cases, it is concluded a nasopharyngeal swab is an optimal sample for detection of respiratory tract viral infection.

## Key points

1. Lung tissue alone was not an optimal specimen for detection of viral respiratory tract infection.
2. Nonetheless, all respiratory tract viral infections regarded as notifiable would have been detected from samples of lung tissue.
3. Nasopharyngeal swabs yielded the greatest proportion of respiratory tract virus detection.
4. Use of four respiratory tract samples (nasopharyngeal swab, tracheal swab, lung swab, and lung tissue) detected only one case of infection that would not have been reported from a nasopharyngeal swab alone.
5. A nasopharyngeal swab is an optimal sample for detection of respiratory tract viral infection.
